# In Vitro Activity of Robenidine Analogues NCL259 and NCL265 against Gram-Negative Pathogens

**DOI:** 10.3390/antibiotics11101301

**Published:** 2022-09-23

**Authors:** Hongfei Pi, Henrietta Venter, Cecilia C. Russell, Kelly A. Young, Adam McCluskey, Stephen W. Page, Abiodun D. Ogunniyi, Darren J. Trott

**Affiliations:** 1 Australian Centre for Antimicrobial Resistance Ecology, School of Animal and Veterinary Sciences, Roseworthy Campus, University of Adelaide, Roseworthy, SA 5371, Australia; 2 Health and Biomedical Innovation, Clinical and Health Sciences, University of South Australia, Adelaide, SA 5000, Australia; 3 Chemistry, School of Environmental and Life Science, University of Newcastle, University Drive, Callaghan, NSW 2308, Australia; 4 Neoculi Pty, Ltd., Burwood, VIC 3125, Australia

**Keywords:** multidrug resistance, Gram-negative pathogens, NCL259, NCL265, efflux pumps, phenylalanine-arginine-beta-naphthylamide, polymyxin B, minimum inhibitory concentration

## Abstract

Multidrug-resistant (MDR) Gram-negative pathogens, especially *Acinetobacter baumannii, Pseudomonas aeruginosa, Escherichia coli* and *Enterobacter* spp., are recognized by the World Health Organization as the most critical priority pathogens in urgent need of drug development. In this study, the in vitro antimicrobial activity of robenidine analogues NCL259 and NCL265 was tested against key human and animal Gram-negative clinical isolates and reference strains. NCL259 and NCL265 demonstrated moderate antimicrobial activity against these Gram-negative priority pathogens with NCL265 consistently more active, achieving lower minimum inhibitory concentrations (MICs) in the range of 2–16 µg/mL. When used in combination with sub-inhibitory concentrations of polymyxin B to permeabilize the outer membrane, NCL259 and NCL265 elicited a synergistic or additive activity against the reference strains tested, reducing the MIC of NCL259 by 8- to 256- fold and the MIC of NCL265 by 4- to 256- fold. A small minority of *Klebsiella* spp. isolates (three) were resistant to both NCL259 and NCL265 with MICs > 256 µg/mL. This resistance was completely reversed in the presence of the efflux pump inhibitor phenylalanine-arginine-beta-naphthylamide (PAβN) to yield MIC values of 8–16 µg/mL and 2–4 µg/mL for NCL259 and NCL256, respectively. When NCL259 and NCL265 were tested against wild-type *E. coli* isolate BW 25113 and its isogenic multidrug efflux pump subunit AcrB deletion mutant (∆AcrB), the MIC of both compounds against the mutant ∆AcrB isolate was reduced 16-fold compared to the wild-type parent, indicating a significant role for the AcrAB-TolC efflux pump from Enterobacterales in imparting resistance to these robenidine analogues. In vitro cytotoxicity testing revealed that NCL259 and NCL265 had much higher levels of toxicity to a range of human cell lines compared to the parent robenidine, thus precluding their further development as novel antibiotics against Gram-negative pathogens.

## 1. Introduction

Multidrug-resistant (MDR) pathogens, especially the ESKAPE organisms (*Enterococcus faecalis/faecium, Staphylococcus aureus, Klebsiella pneumoniae, Acinetobacter baumannii, Pseudomonas aeruginosa* and *Enterobacter* spp.), are a major threat to public health [1]. In particular, MDR Gram-negative critical priority pathogens recognized by the WHO tend to cause high morbidity and mortality (ranging from 30% to 70%) [2,3,4,5] and are a major cause of hospital-acquired infections, including respiratory tract, urinary tract and post-surgical infections [6,7,8]. However, treatment options are limited against these infections, increasing the financial costs to the health systems of many countries [9,10].

The discovery of new antimicrobials has not kept pace with resistance development during the last few decades. The increasing incidence of MDR Gram-negative bacterial infections is associated with many development and post-approval challenges that have resulted in a significant decline of investment by the pharmaceutical industry [11,12,13,14]. Therefore, treatments for MDR critical priority pathogens in particular are now limited, and few novel classes of antimicrobials with a Gram-negative spectrum of activity have progressed through the drug development pipeline [15,16]. Additionally, the presence of an outer membrane in Gram-negative bacteria acts as a substantial barrier to antibiotic uptake, further reducing treatment options, especially for *K. pneumoniae A. baumannii, P. aeruginosa* and *Escherichia coli* (KAPE) infections [17,18,19].

Robenidine (NCL812) is a licensed veterinary anticoccidial agent that has been used globally since the early 1970s in poultry and rabbits [20]. Recently, our laboratory reported that robenidine had antimicrobial activity against methicillin-resistant *Staphylococcus aureus* (MRSA), vancomycin-resistant enterococci (VRE) and *Streptococcus pneumoniae* but limited activity against Gram-negative pathogens [21,22]. However, robenidine in combination with ethylenediaminetetraacetic acid (EDTA), polymyxin B (PMB) or polymyxin B nonapeptide (PMBN) demonstrated potent activity against a broad range of Gram-negative pathogens and showed potential for incorporation into topical and otic formulations for animal or human use [23,24]. Previously, our laboratory reported that an amino pyrimidine analogue of robenidine NCL195 (4,6–bis(2–((E)–4–methylbenzylidene)hydrazinyl)pyrimidin–2–amine) was active against MRSA, VRE and *S. pneumoniae*. We have hypothesized that this Gram-positive specificity is likely due to reduced access to and permeabilization of the cytoplasmic membrane of *S. pneumoniae*, VRE and *S. aureus* by NCL195, thereby hindering the establishment and maintenance of essential energy sources for cell functioning [22]. In the presence of sub-inhibitory concentrations of Gram-negative outer membrane permeabilizers (EDTA, PMB and PMBN), NCL195 was bactericidal against reference KAPE organisms [25]. While the exact mechanism or site of action of robenidine or its analogues has yet to be elucidated, we have used membrane potential measurements and transmission electron microscopy studies to indicate that robenidine and its analogues act by dissipating the membrane potential of Gram-positive and inner membrane potential of Gram-negative organisms [22,25,26,27].

We have been working to further modify the robenidine pharmacophore by synthesizing analogues with improved solubility and potency against ESKAPE pathogens. Among the derivatives, monomeric analogues NCL259 and NCL265 showed the best activity against Gram-negative strains in initial screening tests. Minimum inhibitory concentration (MIC) and minimum bactericidal concentration (MBC) testing showed that concentrations ranging from 8–16 µg/mL inhibited the growth/killed *E. coli*-type strains ATCC 11229 and ATCC 25922 [28,29]. Additionally, these two compounds demonstrated excellent bactericidal activity against *E. coli* in kill kinetics experiments, achieving 100% reduction of colony forming units within 20 min at the MIC for *E. coli* ATCC 25922. In the present study, we further evaluated the in vitro antimicrobial activity of NCL259 and NCL265 against a wider range of Gram-negative isolates (including efflux deficient mutants and clinical isolates from animal infections) as well as screen for in vitro cytotoxicity.

## 2. Materials and Methods

### 2.1. Antimicrobial Agents

**Figure 1:**
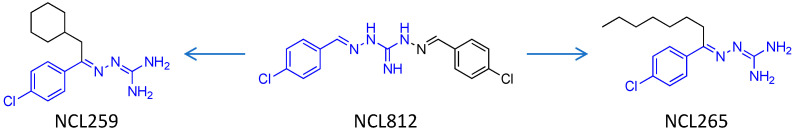
The chemical structures of (*E*)-2-(1-(4-chlorophenyl)-2-cyclohexylethylidene)hydrazine-1-carboximidamide (NCL259) and (*E*)-2-(1-(4-chlorophenyl)heptylidene)hydrazine-1-carboximidamide (NCL265). Blue colouring indicates the common core in NCL259 and NCL265 retained from NCL812.

Analytical grade NCL259 and NCL265, which both contain single benzene rings rather than the double benzene rings joined by the guanidine core of NCL812 (Figure 1) [28] were synthesized at the University of Newcastle (Callaghan, NSW, Australia) and stored in a sealed sample container protected from direct light at 4 °C at the study site (Clinical and Health Sciences, University of South Australia, Adelaide, SA, Australia). Phe-Arg β-naphtyhlamide dihydrochloride cathepsin substrate (PAβN), PMB, dimethyl sulfoxide (DMSO), ampicillin and erythromycin were purchased from Sigma-Aldrich (Macqurie Park, NSW, Australia). Stock solutions of NCL259, NCL265, PAβN, PMB and erythromycin (25.6 mg/mL of each drug) were prepared and stored in 1 mL aliquots at −80 °C and defrosted immediately before use.

### 2.2. Bacterial Strains and Growth Conditions

A variety of Gram-negative reference strains (*E. coli* ATCC 25922, *E. coli* ATCC 10763, *P. aeruginosa* PAO1, *P. aeruginosa* ATCC 27853, *A. baumannii* ATCC 12457, *A. baumannii* ATCC 19606, *K. pneumoniae* ATCC 4352 and *K. pneumoniae* ATCC 33495) were used for initial screening and combination testing with PMB. The organisms were speciated using biochemical testing and MALDI-TOF mass spectrometry (Bruker, Preston, VIC, Australia). Activity testing of NCL259 and NCL265 was also carried out on 51 extraintestinal clinical *E. coli* isolates from companion animals [30], and 83 porcine and bovine enterotoxigenic *E. coli* (ETEC) isolates from the Australian Centre for Antimicrobial Resistance Ecology collection [31]. *E. coli* wild-type (BW 25113) and its mutant BW 25113∆AcrB, in which the inner membrane component AcrB of the tripartite multidrug efflux system AcrA/AcrB/TolC has been deleted [32,33,34], were obtained from the bacterial collection held at Health and Biomedical Innovation, Clinical and Health Sciences, The University of South Australia. All isolates were cultured on sheep blood agar (SBA) plates at 37 °C for 18 h before being subcultured into Luria Bertaini (LB) (Oxoid, Scoresby, VIC, Australia) broth and grown to *A*_600nm_ = 0.5 (equivalent to approx. 1.5 × 10^8^ colony-forming units (CFU)/mL).

### 2.3. Antimicrobial Susceptibility Testing

MICs for NCL259 and NCL265 (serial two-fold dilutions commencing at 256 µg/mL) were determined (in duplicate) in round bottom 96–well microtitre trays (Sarstedt 82.1582.001), using the broth micro-dilution method recommended by the Clinical and Laboratory Standards Institute (CLSI) [35,36]. We have shown previously that robenidine can chelate calcium ions from cation-adjusted Mueller-Hinton broth, which causes the loss of activity of robenidine. Therefore, LB broth was used in this study. Additionally, considering the low solubility of robenidine and its analogues in aqueous solutions, two-fold serial dilutions of stock solutions of these compounds were prepared in 100% DMSO [21]. The MIC for ampicillin against each isolate was determined for each test to serve as an internal quality control. The MICs of isolates were determined by visual reading and using an EnSpire Multimode Plate Reader 2300 at *A*_600nm_.

### 2.4. Synergy Testing by a Checkerboard Assay

MICs of NCL259 or NCL265 in the presence of the efflux pump inhibitor, PAβN were determined using a checkerboard assay. Only strains that were resistant to NCL259 or NCL265 (MIC ≥ 256 μg/mL) were subjected to the checkerboard assay in the presence of PAβN starting at 32 μg/mL using the broth dilution method, as described previously [23,37]. Briefly, a two-fold serial dilution of each antimicrobial solution was prepared in its appropriate solvent (e.g., DMSO for robenidine analogues and Milli-Q water for PAβN), and 1 μL of each dilution was added from wells 12 to 3 (12.8–0.25 μg/mL for robenidine analogues, and 32–2 μg/mL for PAβN). Thereafter, 89 μL of LB broth was added to each well followed by the addition of 10 μL of bacterial suspension (1–5 × 10^6^ CFU/mL) to each well. In this manner, the final concentration of DMSO in each well was 1%. The plate was subsequently incubated at 37 °C for 24 h. The resazurin assay was used for assessment of drug susceptibility of NCL259 and NCL265 (Supplementary Table S1–S6) [38]. The same method was also used to test the MICs of NCL259 or NCL265 in the presence of PMB against a range of Gram-negative reference strains (*E. coli* ATCC 25922, *E. coli* ATCC 10763, *P. aeruginosa* PAO1, *P. aeruginosa* ATCC 27853, *A. baumannii* ATCC 12457, *A. baumannii* ATCC 19606, *K. pneumoniae* ATCC 4352 and *K. pneumoniae* ATCC 33495). Stock solutions were prepared prior to use (25.6–0.25 mg/mL for robenidine analogues; 12.8–0.25 mg/mL for PMB). The interaction of two antibiotics was calculated as the fractional inhibitory concentration index (FICI) using the following formula:

(1) $$\mathrm{FICI}=\frac{{\mathrm{MIC}}_{A\;\mathrm{in}\;\mathrm{combination}}}{{\mathrm{MIC}}_{A\;\mathrm{alone}}}+\frac{{\mathrm{MIC}}_{B\;\mathrm{in}\;\mathrm{combination}}}{{\mathrm{MIC}}_{B\;\mathrm{alone}}}$$

where A is NCL259 or NCL265 and B is PAβN or PMB. According to FICI calculations, the interaction between two compounds were interpreted as follows: synergistic (FICI ≤ 0.5); additive or partially synergistic (0.5 < FICI ≤ 1); indifferent (1 < FICI ≤ 4); and antagonistic (FICI > 4), as described previously [23,25,39]. The dose reduction index (DRI) was used to describe the difference between the effective doses of NCL259 or NCL265 in combination with PMB or PAβN in comparison to their individual doses, using the following formula:

(2) $$\mathrm{DRI}=\frac{{\mathrm{MIC}}_{A\;\mathrm{alone}}}{{\mathrm{MIC}}_{A\;\mathrm{in}\;\mathrm{combination}}}$$

Evaluation of DRI is important clinically because it can allow the dose of a potentially toxic drug to be lowered in combination with another compound without reducing the drug’s efficacy. A DRI higher than 1 is viewed as beneficial [23,25,39].

### 2.5. In Vitro Cytotoxicity and Hemolysis Assays

Potential toxicity of NCL259 and NCL265 to mammalian cells relative to the parent compound (NCL812) was examined using Caco-2 (human colorectal adenocarcinoma cell line), HEL 299 (non-cancerous human lung fibroblast cell line), Hep G2 (human hepatocellular carcinoma cell line), MCF-7 (human mammary gland adenocarcinoma cell line), MDBK (normal Madin–Darby bovine kidney cell line) and Vero (normal adult African green monkey cell line), essentially as described previously [22]. Toxicity to the cell lines was performed using serial two-fold dilutions of each compound from 128 to 1 μg/mL in duplicates in flat bottom 96 well tissue culture trays (Sarstedt 83.3924) containing approximately 2 × 10^4^ cells per well in antibiotic-free Dulbecco’s Modified Eagle’s Medium (DMEM; Gibco Cat No: 12430; Thermo Fisher Scientific, Scoresby, VIC, Australia) consisting of 10% (*v/v*) fetal bovine serum at 37 °C, 5% CO_2_ for 24 h. Duplicate wells containing ampicillin at 128 to 1 μg/mL were used as controls. Thereafter, 10% (*v/v*) of the WST-1 cell proliferation assay reagent (Cat No: 05015944001; Roche Life Science, North Ryde, NSW, Australia) was added to each well, the trays were further incubated at 37 °C, 5% CO_2_ for 1 h and absorbance read at *A*_450nm_ on a Multiskan Ascent 354 Spectrophotometer (Labsystems, Lilydale, VIC, Australia). The inhibitory concentration of each compound was determined as the half maximal inhibitory concentration (IC_50_) at *A*_450nm_.

The haemolytic activity of the compounds was determined using 1% sheep red blood cells (SRBCs), as described previously [22]. Duplicate wells containing ampicillin at 128 to 1 μg/mL, 1% Triton X (for 100% lysis) or phosphate-buffered saline (pH 7.0) were used as controls. After incubation at 37 °C with shaking at 100 rpm for 1 h, the trays were centrifuged at 1000× *g* for 3 min and 100 μL of supernatant from each well was transferred into a new 96-well tray. Absorbance was measured at *A*_450nm_ on a Multiskan Ascent 354 Spectrophotometer (Labsystems) and plotted against each dilution. The haemolytic titre (HC_50_) of each compound was determined as the reciprocal of the dilution at which 50% of SRBCs were lysed at *A*_450nm_.

## 3. Results

### 3.1. NCL259 and NCL265 Show Antimicrobial Activity against Gram-Negative Reference Strains

We previously demonstrated the activity of NCL259 and NCL265 against a panel of human Gram-negative isolates comprising 19 *E. aerogenes*, 20 *E. coli*, 19 *E. cloacae*, 20 *K. pneumoniae* and 16 *K. oxytoca*, returning MIC ranges of 2–64 μg/mL for both compounds against these isolates. Additionally, in a time-kill kinetics assay, we reported that both NCL259 and NCL265 were bactericidal (>5 log 10 reduction in CFU) against *E. coli* ATCC 25922 and *K. pneumoniae* ATCC 4352, after 30 min treatment [28]. In the present study, we extended the range of activity of NCL259 and NCL265 against more Gram-negative reference strains including *E. coli* ATCC 25922, *E. coli* ATCC 10763, *P. aeruginosa* PAO1, *P. aeruginosa* ATCC 27853, *A. baumannii* ATCC 12457, *A. baumannii* ATCC 19606, *K. pneumoniae* ATCC 4352 and *K. pneumoniae* ATCC 33495). Again, antimicrobial activity was variable with an MIC range of 2–64 μg/mL demonstrated for both analogues (Table 1), in agreement with our previous findings. Notably, MICs were several fold lower for the *Acinetobacter* isolates compared to *E. coli*.

### 3.2. Combination of NCL259 or NCL265 with PMB Increases Antimicrobial Activity against Gram-Negative Reference Strains

PMB is a Gram-negative outer membrane permeabilizer [40,41]; therefore, we hypothesized that the disruption in the Gram-negative outer membrane by PMB would allow access to the test compounds’ site of action, thereby increasing their antimicrobial activity. As such, the effect of a combination of NCL259 or NCL265 with sub-inhibitory concentrations of PMB against a range of Gram-negative reference strains (*E. coli* ATCC 25922, *E. coli* ATCC 10763, *P. aeruginosa* PAO1, *P. aeruginosa* ATCC 27853, *A. baumannii* ATCC 12457, *A. baumannii* ATCC 19606, *K. pneumoniae* ATCC 4352 and *K. pneumoniae* ATCC 33495) was investigated. We showed through FICI and DRI calculations that the combination of either NCL259 or NCL265 with PMB is synergistic or additive against the strains tested (Table 1).

### 3.3. NCL259 and NCL265 Show Antimicrobial Activity against Gram-Negative Clinical Isolates from Animals

The activity of NCL259 and NCL265 was tested against 51 clinical extraintestinal pathogenic *E. coli* (ExPEC) isolates from companion animals and 83 porcine and bovine ETEC isolates. NCL259 demonstrated antimicrobial activity at a range of 4–64 μg/mL, while NCL265 appeared to be more potent, with activity at a range of 2–16 μg/mL (Table 2).

### 3.4. Efflux Pump Inhibitor (PAβN) Increases the Antimicrobial Activity of NCL259 and NCL265 against Resistant Klebsiella Isolates

Of all the 147 isolates and reference strains tested in this study, only *K. pneumoniae* 13GNB–429, *K. pneumoniae* 13GNB–550 and *K. oxytoca* 13GNB–582 were resistant to NCL259 and NCL265 (MICs >256 μg/mL). We investigated the possibility that this resistance could be efflux pump-driven by testing a combination of NCL259 or NCL265 with the resistance-nodulation efflux pump inhibitor, PAβN. The combination of PAβN with either NCL259 (Table 3 and Tables S1–S3) or NCL265 (Table 4 and Tables S4–S6) produced a synergistic or additive interaction against the three isolates, returning even lower MICs (8–16 μg/mL for NCL259 and 2–4 μg/mL for NCL265) compared to susceptible isolates.

### 3.5. The AcrA/AcrB/TolC Multidrug Efflux Pump Impacts the Activity of NCL259 and NCL265

Given the findings that PAβN restored the antimicrobial activity of NCL259 and NCL265 against three resistant *Klebsiella* isolates, we further probed the hypothesis that resistance to NCL259 and NCL265 by the *Klebsiella* isolates is driven by the multidrug efflux pump. For this assay, the antimicrobial activity of NCL259 and NCL265 was tested against a deletion mutant of the inner membrane component AcrB subunit (∆AcrB) in the AcrA/AcrB/TolC multidrug efflux pump complex. The MICs of NCL259 and NCL265 were dramatically reduced by 16–fold in the ∆AcrB mutant compared to its otherwise isogenic wild-type *E. coli* parent (Table 5), suggesting a role for this well-recognized MDR efflux system in reducing the activity of NCL259 and NCL265 in Gram-negative organisms.

### 3.6. NCL259 and NCL265 Are Toxic to Mammalian Cells and Hemolytic to Erythrocytes

The in vitro cytotoxicity profiles of NCL259 and NCL265 to a variety of mammalian cells and SRBCs in comparison to the original robenidine (NCL812) were examined with a view to consideration of their potential for further in vivo safety and efficacy testing. The results showed that both compounds were considerably more cytotoxic to all the cell lines tested and lysed SRBCs at very low concentrations when compared to the original parent molecule (Table 6).

## 4. Discussion

Multidrug-resistant KAPE infections have become a worldwide threat causing health and economic problems. However, there have not been any novel mode of action antimicrobials for treatment of Gram-negative bacterial infections introduced to market in the last two decades. Previously, we demonstrated excellent potential of a robenidine (NCL812) analogue (NCL195) as a new pyrimidine-based drug scaffold for the treatment of infections caused by Gram-positive bacteria based on its more favourable pharmacokinetic/pharmacodynamic profiles and reduced toxicity compared to the parent guanidine-based robenidine [22,23,25]. The previous findings also demonstrated in vitro efficacy of NCL812 and NCL195 against Gram-negative organisms in combination with outer membrane permeabilizers (EDTA and PMB) including demonstrated activity against multidrug-resistant strains. In the present study, we further evaluated the in vitro antimicrobial activities of two monomeric guanidine-based analogues (NCL259 and NCL265) that have previously shown promising Gram-negative activity.

The present study had three major findings. First, NCL259 and NCL265 demonstrated antimicrobial activity against a very large range (*n* = 145) of Gram-negative reference strains and isolates from humans and animals, corroborating our earlier findings [28]. Second, use of an efflux pump inhibitor increased the antimicrobial activity of NCL259 and NCL265 against the very few (three) highly resistant *Klebsiella* isolates. Additionally, an AcrB knockout mutant had 16-fold lower MICs compared to the wild-type, indicating a predominant role for efflux-mediated resistance (present in many Gram-negative pathogens) against guanidine analogues. Third, further exploration of Gram-negative activity in vivo was curtailed by increased in vitro cytotoxicity compared to the parent compound, which is likely to be another negative feature preventing further development of monomeric guanidine analogues as novel antimicrobials for Gram-negative infections.

NCL265 (MIC range 2–32 μg/mL) demonstrated more potent antimicrobial activity against a variety of Gram-negative human and animal isolates, including human KAPE pathogens compared to NCL259 (8–64 μg/mL), suggesting that the smaller size of these monomeric robenidine analogues may enable penetration of the Gram-negative outer membrane through porins (thus reaching the likely target site within the bacterial inner membrane) compared to the larger dimeric guanidine and pyrimidine-based structures. As we have previously shown, much improved MICs were obtained for both molecules in the presence of outer membrane permeabilizers, a feature of both the parent compound (NCL812) and previously described dimeric analogues NCL195 and NCL179 [22,23,25,42]. Whilst outer membrane permeabilizers such as EDTA may facilitate transport across the outer membrane through destabilization and loss of lipopolysaccharide chains, outer membrane permeabilizers could also affect microbial efflux systems [43,44].

Efflux pumps are protein complexes located on the cell membrane in both Gram-positive and Gram-negative bacteria. Efflux pumps are one of the main pathways for transporting toxic substrates including many classes of antimicrobials, across the cell membrane [45], thereby conferring multidrug resistance [24]. In Gram-negative bacteria, tripartite protein assemblies that span the inner membrane, the outer membrane and periplasmic space are the clinically relevant efflux pumps [46]. In Enterobacterales such as *E. coli* and *Klebsiella* spp., AcrB is the inner membrane protein of the Resistance-Nodulation-Division (RND) family, AcrA is the membrane adapter protein and TolC is the outer membrane factor protein [47].

The MICs of NCL259 and NCL265 against three resistant strains of *Klebsiella* spp. in in the presence the efflux-pump inhibitor (PAβN) were reduced by 16- to 32-fold and 64- to 128-fold, respectively. These findings confirm a role for efflux-mediated resistance to these smaller robenidine analogues. To provide further evidence to support this hypothesis, the MICs of NCL259 and NCL265 against the mutant ∆AcrB isolate were found to be 16-fold lower compared to the wild-type.

Given our previous findings that the combination of NCL812, NCL195, or NCL179 with Gram negative outer membrane permeabilizers (EDTA and polymyxins) could result in a synergistic or additive activity against Gram-negative bacteria [22,23,25,42], we also tested NCL259 or NCL265 activity in combination with PMB. For the Gram-negative bacteria reference strains tested, either a synergistic or additive interaction was observed, reducing the MIC of NCL259 by 8- to 256-fold and the MIC of NCL265 by 4- to 256-fold.

**Figure 2:**
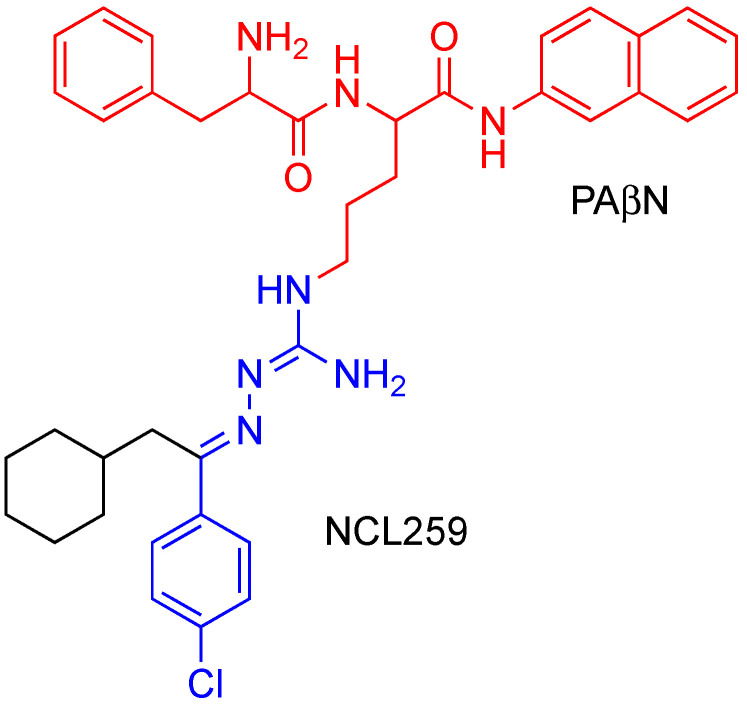
The hypothesized chemical structures of (*E*)-2-(1-(4-chlorophenyl)-2-cyclohexylethylidene)hydrazine-1-carboximidamide (NCL259) and PAβN combined compound.

The original robenidine molecule (NCL812) has been shown to have antimicrobial activity against protozoa [48], fungi [49] and bacteria [21,23] and a level of cytotoxicity that likely restricts its use to orally administered or topically applied formulations. Whilst preliminary studies in *Candida albicans* have suggested a likely mechanism of action resulting in fungal cell membrane lysis, further elucidation of the site and mechanism of action in protozoa and bacteria may aid in understanding the molecular basis of membrane cell lysis in this interesting antimicrobial class with many potential clinical applications. As higher levels of cytotoxicity to a variety of mammalian cell lines and red blood cells were demonstrated for NCL259 and NCL265 compared to the parent molecule, these robenidine analogues are unlikely to be further developed clinically. However, they may be very useful research tools for understanding the basis of selective toxicity in the much larger analogues. Nevertheless, given our findings that addition of PAβN reduced the MIC of NCL259 and NCL265 against resistant *Klebsiella* spp., we hypothesize that a modified analogue combining NCL259 or NCL265 with PAβN could be a potential candidate for future studies, which may provide improved antimicrobial activity with reduced MIC profiles whilst potentially reducing the cytotoxicity associated with each individual compound for future in vivo tests (Figure 2).

## 5. Conclusions

In this study, we demonstrated the in vitro antimicrobial activity of a new antibacterial class represented by NCL259 and NCL265 against a variety of clinical Gram-negative bacteria, including priority antimicrobial-resistant pathogens currently listed by the WHO [50,51]. Whilst synergistic or additive interaction of the combination of NCL259 or NCL265 with sub-inhibitory concentrations of PAβN and PMB was observed, their susceptibility to antimicrobial efflux and high levels of cytotoxicity to multiple cell lines and red blood cells preclude their further advancement to in vivo characterisation. Nevertheless, they may be useful research analogues in ongoing mechanism of action studies, which are required to further improve promising scaffolds such as NCL195 and NCL179.

## Figures and Tables

**Table 1:** MIC (μg/mL) values for PMB, NCL259 and NCL265 and in combination with PMB against Gram-negative reference strains.

Isolates	^1^ MIC (μg/mL)						^2^ Combination Effect (FICI)		^3^ Dose Reduction Index (DRI)	
	Single Drug			Combination						
	PMB	NCL259	NCL265	PMB	NCL259	NCL265	NCL259	NCL265	PMB:NCL259	PMB:NCL265
*E. coli* ATCC 25922	0.5	16	4	0.25	1	1	Additivity (0.563)	Additivity (0.75)	2:16	2:4
*E. coli* ATCC 10763	1	64	64	0.5	0.25	0.25	Additivity (0.504)	Synergism (0.254)	2:256	4:256
*P. aeruginosa* PAO1	1	64	64	0.5	1	0.25	Additivity (0.516)	Additivity (0.504)	2:64	2:256
*P. aeruginosa* ATCC 27853	1	64	32	0.5	2	0.5	Additivity (0.531)	Additivity (0.516)	2:32	2:64
*A.baumannii* ATCC 12457	1	8	2	0.5	1	0.5	Additivity (0.625)	Additivity (0.75)	2:8	2:4
*A.baumannii* ATCC 19606	1	8	2	0.5	1	0.5	Additivity (0.625)	Additivity (0.75)	2:8	2:4
*K. pneumoniae* ATCC 4352	1	8	2	0.25	1	0.5	Synergism (0.375)	Synergism (0.5)	4:8	4:4
*K. pneumoniae* ATCC 33495	1	64	64	0.5	1	2	Additivity (0.516)	Synergism (0.281)	2:64	4:32

^1^ MIC, minimum inhibitory concentration; ^2^ FICI, fractional inhibitory concentration index: synergistic, FICI ≤ 0.5; additive or partially synergistic, 0.5 < FICI ≤ 1; indifferent, 1 < FICI ≤ 4; and antagonistic, FICI > 4; ^3^ DRI, dose reduction index.

**Table 2:** MIC range, MIC_50_, MIC_90_ for NCL259 and NCL265 against companion animal ExPEC, and porcine/bovine ETEC isolates.

Isolates	NCL259			NCL265		
	^1^ MIC Values (μg/mL)			MIC Values (μg/mL)		
	MIC Range	MIC50	MIC90	MIC Range	MIC50	MIC90
Companion animal ^2^ ExPEC (*n* = 51)	16–64	32	32	4–16	32	32
Porcine/bovine ^3^ ETEC (*n* = 83)	4–64	8	8	2–16	8	8

^1^ MIC, minimum inhibitory concentration; ^2^ ExPEC, Extraintestinal Pathogenic *Escherichia coli*. ^3^ ETEC, Enterotoxigenic *Escherichia coli*.

**Table 3:** MICs of NCL259, PAβN and in combination against resistant *K. pneumoniae* and *K. oxytoca* isolates.

Isolates	^1^ MIC (μg/mL)				Combination Effect (^2^ FICI)	^3^ DRI PAβN:NCL259
	Single Drug		Combination			
	PAβN	NCL259	PAβN	NCL259		
*K. pneumoniae* 13GNB–429	>32	>256	16	16	Synergism (0.281)	4:16
*K. pneumoniae* 13GNB–550	>32	>256	32	8	Additivity (0.531)	2:32
*K. oxytoca* 13GNB–582	>32	>256	16	8	Synergism (0.281)	4:32

^1^ MIC, minimum inhibitory concentration; ^2^ FICI, fractional inhibitory concentration index: synergistic, FICI ≤ 0.5; additive or partially synergistic, 0.5 < FICI ≤ 1; indifferent, 1 < FICI ≤ 4; and antagonistic, FICI > 4; ^3^ DRI, dose reduction index.

**Table 4:** MICs of NCL265 and PAβN and in combination against resistant *K. pneumoniae* and *K. oxytoca* isolates.

Isolates	^1^ MIC (μg/mL)				Combination Effect (^2^ FICI)	^3^ DRI PAβN:NCL265
	Single Drug		Combination			
	PAβN	NCL265	PAβN	NCL265		
*K. pneumoniae* 13GNB–429	>32	>256	32	4	Additivity (0.516)	2:64
*K. pneumoniae* 13GNB–550	>32	>256	32	4	Additivity (0.516)	2:64
*K. oxytoca* 13GNB–582	>32	>256	32	2	Additivity (0.508)	2:128

^1^ MIC, minimum inhibitory concentration; ^2^ FICI, fractional inhibitory concentration index: synergistic, FICI ≤ 0.5; additive or partially synergistic, 0.5 < FICI ≤ 1; indifferent, 1 < FICI ≤ 4; and antagonistic, FICI > 4; ^3^ DRI, dose reduction index.

**Table 5:** MICs of NCL259 and NCL265 against efflux-pump mutant and wild-type *E. coli* isolates.

Isolates	^1^ MIC Values (μg/mL)		
	NCL259	NCL265	Erythromycin
Wild-type (*E. coli* BW 25113)	64	16	>128
*E. coli* BW 25113 (∆AcrB)	4	1	2

^1^ MIC, minimum inhibitory concentration. Erythromycin was used as a positive control in the MIC test.

**Table 6:** IC_50_ and HC_50_ for NCL812, NCL259 and NCL265 against a variety of mammalian cell lines.

Cell Line	^1^ IC_50_ or ^2^ HC_50_ Values (μg/mL) ^3^ for:			
	NCL812	NCL259	NCL265	Ampicillin
^1^ Caco-2	8, 12	<1, 3	<1, <2	>128, >128
^1^ HEL 299	8, 6	<2, <2	<2, <2	>128, >128
^1^ Hep G2	6, 8	<1, <1	<1, <1	>128, >128
^1^ MCF-7	8, 12	3, 6	<1, 6	>128, >128
^1^ MDBK	8, 12	2, 2	3, 2	>128, >128
^1^ Vero	8, 4	6, 3	6, 3	>128, >128
^2^ SRBC	32, 32	16, 32	16, 32	>128, >128

^1^ IC_50_ = Inhibitory concentration; determined as the half maximal inhibitory concentration of each compound at *A*_450nm_. ^2^ HC_50_ = Haemolytic titre; determined as the reciprocal of the dilution at which 50% of SRBCs were lysed at *A*_450nm_. ^3^ Values are from two independent experiments. Caco-2 (human colorectal adenocarcinoma cell line); HEL 299 (non-cancerous human lung fibroblast cell line); Hep G2 (human hepatocellular carcinoma cell line); MCF-7 (human mammary gland adenocarcinoma cell line); MDBK (normal Madin–Darby bovine kidney cell line); Vero (normal adult African green monkey cell line); SRBCs (sheep red blood cells).

## Data Availability

The data presented in this study are available from the corresponding authors upon request. The data are not publicly available due to privacy and access restrictions.

## Supplementary Materials

The following supporting information can be downloaded at https://www.mdpi.com/article/10.3390/antibiotics11101301/s1, Table S1. NCL259 in combination with PAβN against *K. pneumoniae* 13GNB-429; Table S2. NCL259 in combination with PAβN against *K. pneumoniae* 13GNB-550; Table S3. NCL259 in combination with PAβN against *K. oxytoca* 13GNB-582; Table S4. NCL265 in combination with PAβN against *K. pneumoniae* 13GNB-429; Table S5. NCL265 in combination with PAβN against *K. pneumoniae* 13GNB-550; Table S6. NCL265 in combination with PAβN against *K. oxytoca* 13GNB-582.
