# Supplementary material for: In Vitro Activity of Robenidine Analogues NCL259 and NCL265 against Gram-Negative Pathogens
Source: Antibiotics (Basel). 2022 Sep 23;11(10):1301. doi: 10.3390/antibiotics11101301 (PMC9598656; doi:10.3390/antibiotics11101301)
Supplement: Supplementary file 1 [file antibiotics-11-01301-s001.zip › antibiotics-1921122-supplementary.pdf]

Table S1. NCL259 in combination with PAβN against *K. pneumoniae* 13GNB-429.

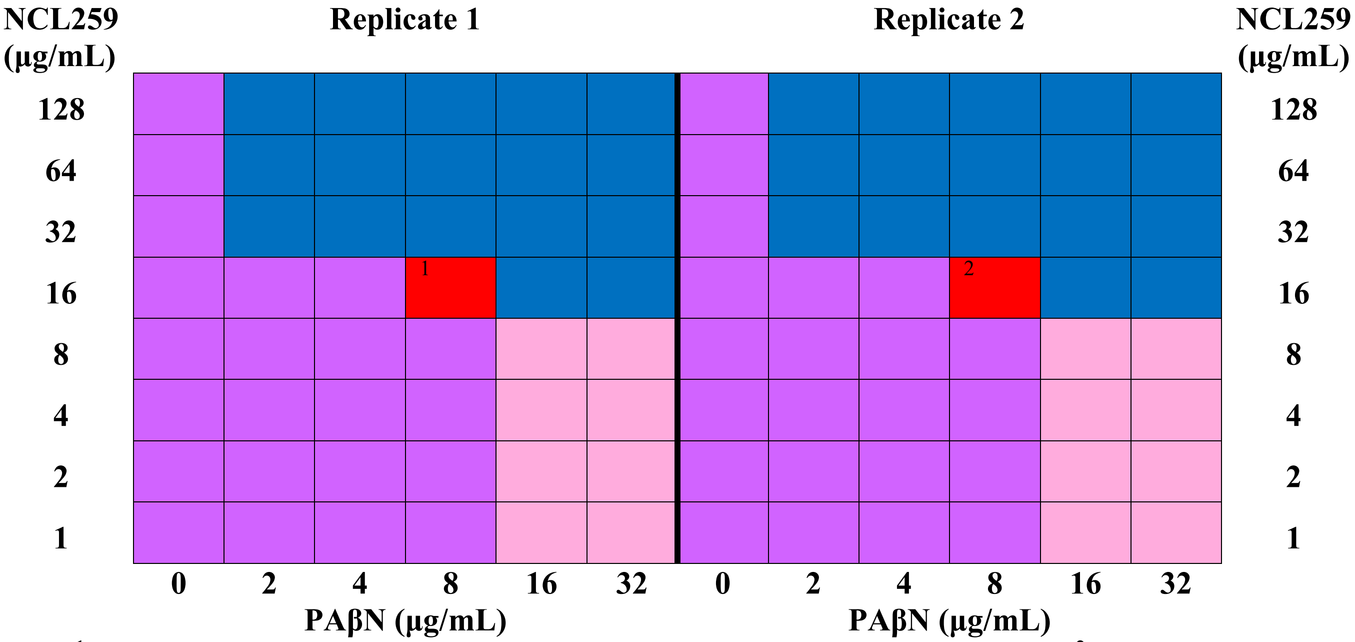

[1] The synergistic interaction of NCL259 at 16 µg/mL and PAβN at 8 µg/mL; [2] the synergistic interaction of NCL259 at 16 µg/mL and PAβN at 8 µg/mL in replicate. Blue and pink indicate the absence or presence of living colonies, respectively.

Table S2. NCL259 in combination with PAβN against *K. pneumoniae* 13GNB-550.

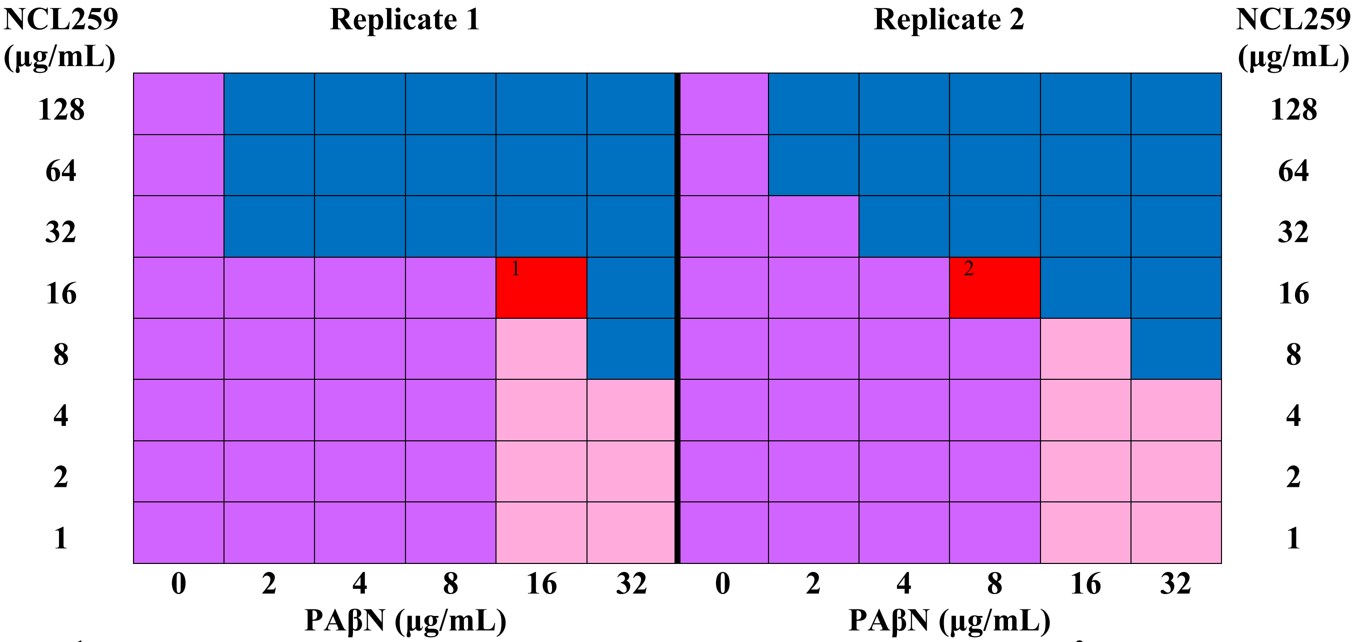

[1] The addictive interaction of NCL259 at 16 µg/mL and PAβN at 16 µg/mL; [2] the synergistic interaction of NCL259 at 16 µg/mL and PAβN at 8 µg/mL in replicate. Blue and pink indicate the absence or presence of living colonies, respectively.

Table S3. NCL259 in combination with PAβN against *K. oxytoca*13GNB-582.

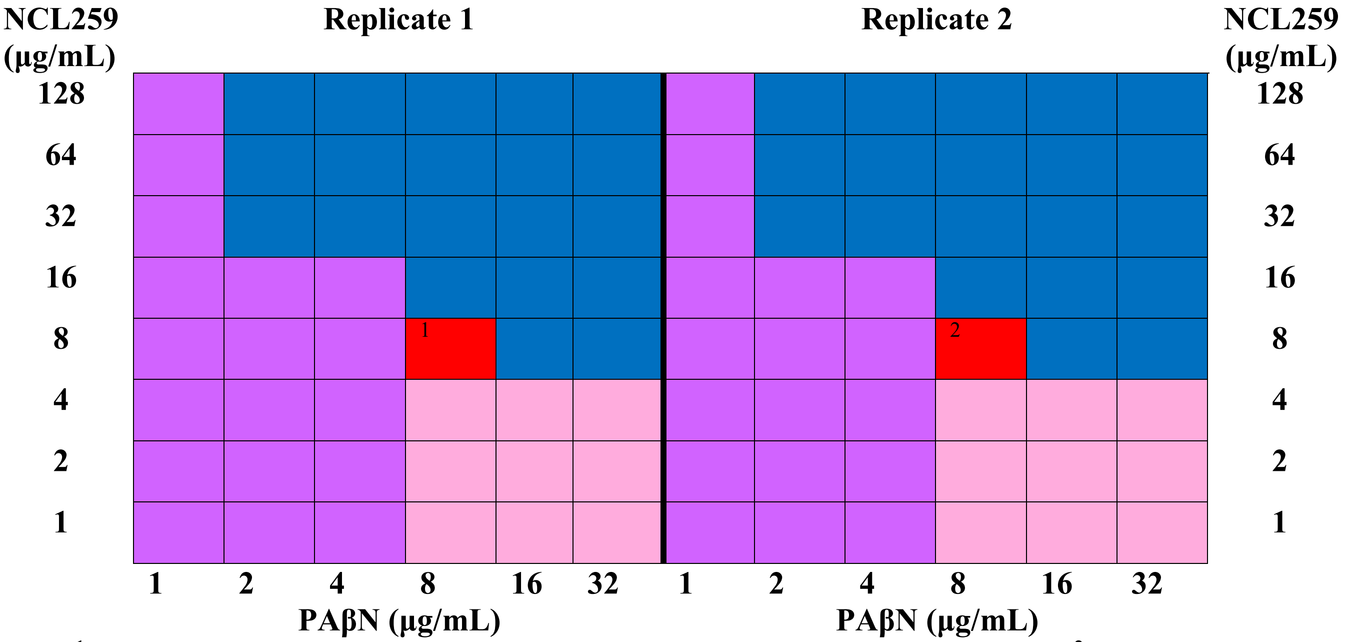

[1] The synergistic interaction of NCL259 at 8 µg/mL and PAβN at 8 µg/mL; [2] the synergistic interaction of NCL259 at 8 µg/mL and PAβN at 8 µg/mL in replicate. Blue and pink indicate the absence or presence of living colonies, respectively.

Table S4. NCL265 in combination with PAβN against *K. pneumoniae* 13GNB-429.

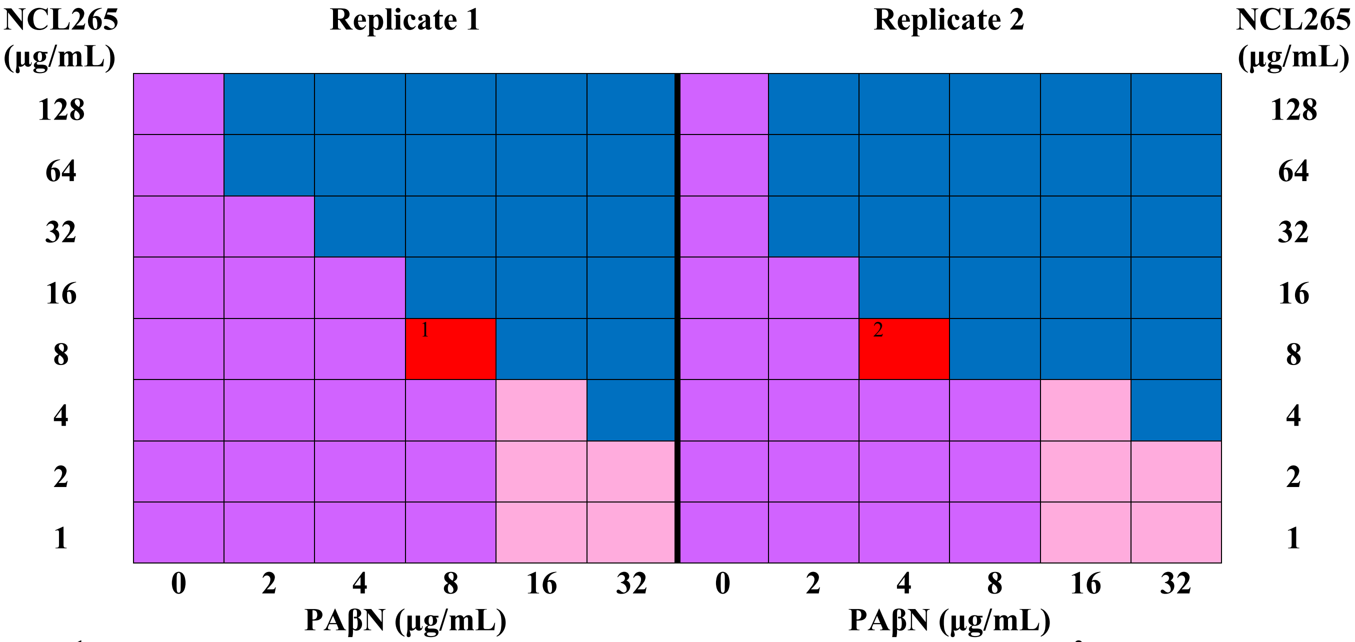

[1] The synergistic interaction of NCL265 at 8 µg/mL and PAβN at 8 µg/mL; [2] the synergistic interaction of NCL265 at 8 µg/mL and PAβN at 4 µg/mL in replicate. Blue and pink indicate the absence or presence of living colonies, respectively.

Table S5. NCL265 in combination with PAβN against *K. pneumoniae* 13GNB-550.

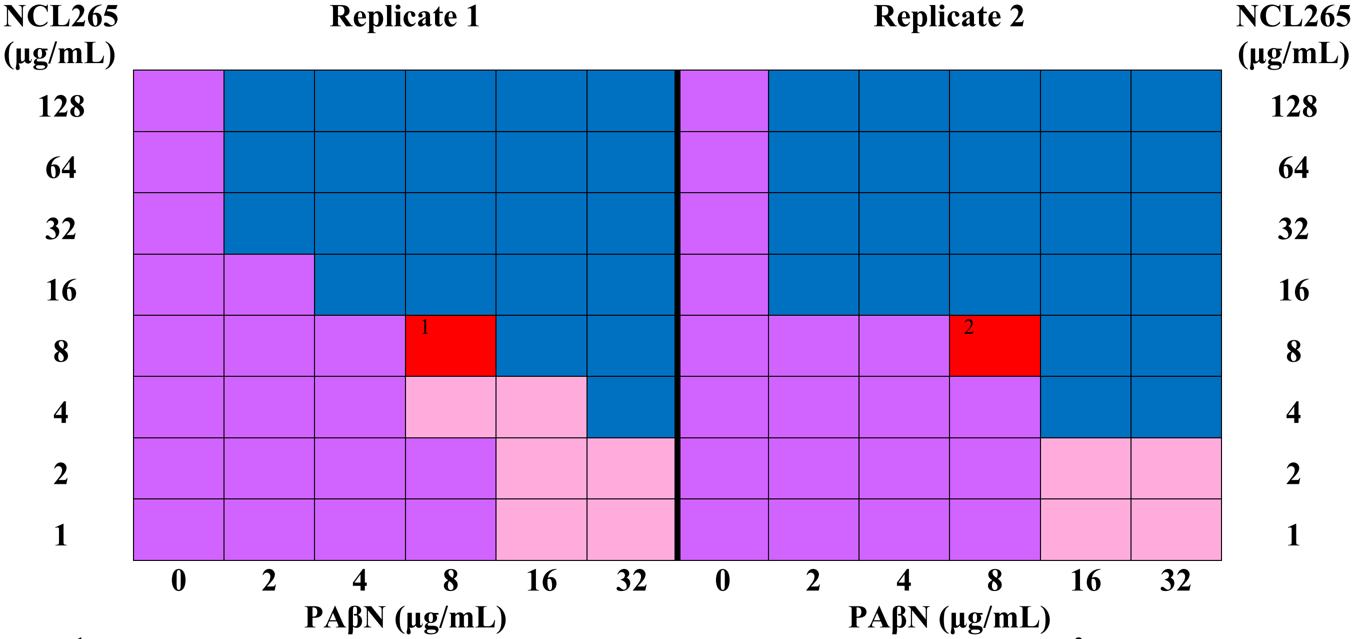

[1] The synergistic interaction of NCL265 at 8 μg/mL and PAβN at 8 μg/mL; [2] the synergistic interaction of NCL265 at 8 μg/mL and PAβN at 8 μg/mL in replicate. Blue and pink indicate the absence or presence of living colonies, respectively.

Table S6. NCL265 in combination with PAβN against *K. oxytoca*13GNB-582.

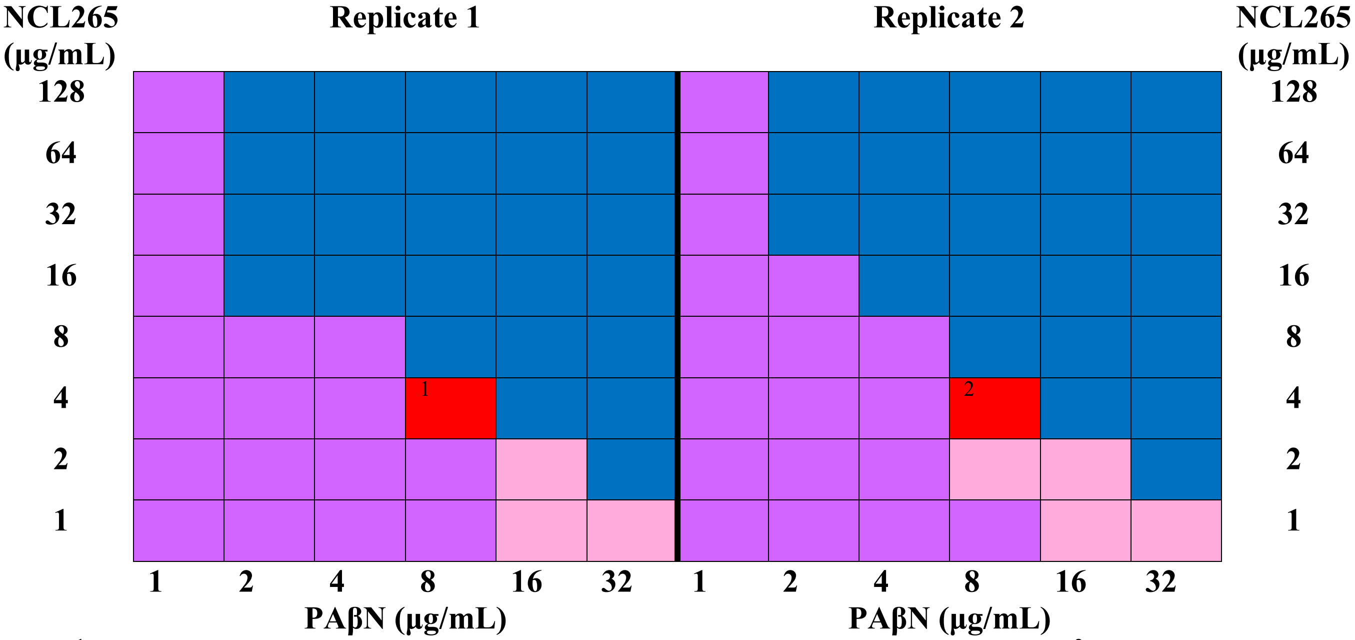

[1] The synergistic interaction of NCL265 at 4 μg/mL and PAβN at 8 μg/mL; [2] the synergistic interaction of NCL265 at 8 μg/mL and PAβN at 8 μg/mL in replicate. Blue and pink indicate the absence or presence of living colonies, respectively.
